# Esophageal cancer: a twenty-four-year experience at a tertiary care center with an evaluation of the prognostic significance of the neutrophil-lymphocyte ratio

**DOI:** 10.1186/s12876-023-03115-5

**Published:** 2024-01-09

**Authors:** Walaa Mahmoud, Lara Hassoun, Anthony Kerbage, Deborah Mukherji, Ali Shamseddine, Sally Tamraz, Ayman Hakim, Kassem Barada

**Affiliations:** 1 Division of Gastroenterology and Hepatology, American University of Beirut Medical Center, Beirut, Lebanon; 2 Division of Gastroenterology and Hepatology, Mayo Clinic, Rochester, MN United States of America

**Keywords:** Esophagus, Cancer, Lymphocyte, Survival

## Abstract

**Background:**

A high neutrophil-lymphocyte ratio (NLR) may be associated with worse survival in esophageal cancer (EC). Our aims were to describe the demographic and clinical data of EC in a tertiary referral center in Lebanon and to determine the prognostic value of NLR.

**Methods:**

A retrospective cohort study based on chart review of patients diagnosed with EC was conducted at the American University of Beirut Medical Center (AUBMC). The demographic characteristics, clinical presentation and outcomes were described and compared between squamous cell carcinomas (ESCC) and adenocarcinomas (EAC). Data about esophageal cancer incidence were obtained from the National Cancer Registry, the Ministry of Public Health and GLOBOCAN 2020. Cox regression analysis was performed to determine whether the NLR is an independent predictor of survival, using variables based on clinical knowledge and previously established data.

**Results:**

110 patients were diagnosed with EC, which was the least common among other gastrointestinal malignancies. Our follow up rates reached 86.4%. The median survival was 9 months (IQR 3–25.5.) and was comparable between ESCC (median of 7 months, IQR 2–25) and EAC (median of 9 months, IQR 3–26.3), *p* = 0.803. Advanced stage was associated with a worse prognosis (*p* = 0.037). The mean NLR(±SD) was 5.20 ± 6.8, with no significant difference between EAC and ESCC (4.5 ± 3.4 vs. 5.9 ± 9.2, *p* = 0.420) or between early or advanced stages (5.4 ± 8.1 vs. 4.7 ± 6.8, *p* = 0.732). The area under the curve for the NLR was 0.560 (95% CI: 0.374–0.746, *p* = 0.488). After adjusting for age, gender, TNM staging and grading, cox regression analysis showed that an increased NLR was a significant predictor of mortality, with an adjusted hazard ratio of 1.095 (*p* = 0.011).

**Conclusion:**

EC is quite uncommon in Lebanon despite a high prevalence of smoking and obesity. Advanced stage and high NLR were associated with a negative prognostic value.

## Introduction

Esophageal cancer (EC) remains a rapidly growing international concern. According to GLOBOCAN 2020, it is among the eight most common cancers reaching 3.1% of all cancer incidence globally [1]. Despite advancement in diagnosis and treatment, it is still associated with an increasing overall incidence and mortality. It is now considered the sixth leading cause of cancer mortality worldwide, accounting for 5% of all cancer deaths annually [2]. The 5 year survival rates are reported to be in the range of 15–25% [3].

A wide geographic variation in incidence is characteristic of EC, with the highest found in Southern and Eastern Africa and Eastern Asia and lowest in Western and Middle Africa and Central America [4, 5]. Only few studies have tackled the epidemiology of EC in Middle Eastern countries including Lebanon. According to the Cancer Incidence in Five Continents and GLOBOCAN 2020, the age adjusted incidence rate of EC in Lebanon was 0.77 per 100,000 males and 0.36 per 100,000 females, which was the lowest among other countries with a similar geographic location [6].

Despite its low incidence, mortality continues to be a challenge for all types of esophageal cancer. When looking into the possible prognostic factors for this deadly disease, the neutrophil to lymphocyte ratio (NLR) has emerged as a potential marker for various diseases including EC, other cancers and infections.

In this retrospective study, we aim to describe the demographic and clinical data on EC at a major referral center in Lebanon and to look at the survival rate with respect to different factors including the shift in therapeutic interventions that took place in 2010. We also study the NLR and its possible significance as a standard prognostic factor.

## Patients and methods

### Study design

We conducted a single institution retrospective cohort study based on chart review of all cases of EC cases seen at AUBMC, a tertiary referral center, between January 1995 and December 2019. A list of patients with EC was acquired from the Department of Medical Records at AUBMC. Patients who had benign esophageal tumors were excluded. The study was approved by the local Institutional Review Board committee at AUBMC (Protocol Number: BIO-2020-0001). The IRB ensures all human subject research conducted by AUB investigators complies with the laws of Lebanon, AUB policies, U.S. Federal regulations governing human subject research, and, when applicable, US Federal laws. The IRB ensures awareness of and respect for the rights of human research subjects.

### Data collection

Patients’ demographics, clinical information, risk factors, tumor characteristics and management history were collected from medical records, clinical charts and hospital files. The collected data included nationality, age at diagnosis, gender, residence, body mass index (BMI), smoking and alcohol use, pre-existing esophageal and gastric disease, presenting symptoms, pathologic, radiologic and endoscopic evaluation, management plan as well as the last day of follow up and date of death whenever available. The TNM staging system was used. BMI was categorized in accordance with WHO standards; subjects were categorized into normal (BMI < 25), overweight (BMI 25–30) or obese: (BMI ≥ 30 kg/m2). Lab tests including blood neutrophil and lymphocyte counts were then taken from the patients’ records during their first admission or first clinic follow up, upon diagnosis.

Data about esophageal cancer incidence were obtained from the National Cancer Registry, the Ministry of Public Health and GLOBOCAN 2020.

### Statistical analysis

SPSS 23.0 (IBM, Armonk, NY) was used for statistical analysis. First, univariate analyses were carried out by calculating the number and percent for categorical variables, and the mean and standard deviation (SD) for the continuous ones. Pearson- chi squared test was performed to compare distribution of categorical variables between ESCC and EAC via cross-tabulations. Fisher’s exact test was used, instead of Pearson chi square test, in case a category within a certain variable had less than 5% of the sample studied. A multivariate analysis was then performed to determine factors that might affect the survival of esophageal cancer patients, including the NLR. We evaluated the NLR across different types of esophageal cancers and tumor grades and stages. We employed Receiver Operating Characteristic (ROC) curve analysis to assess the NLR’s prognostic value in predicting end-of-follow-up mortality, calculating the area under the curve (AUC) along with its 95% Confidence Interval (CI) and associated *p*-value. Initially, a Cox regression model was performed, incorporating all variables traditionally associated with mortality based on clinical knowledge and previously established data. Subsequently, a refined Cox regression analysis was done, applying more stringent statistical criteria, which involved the exclusion of patients lacking follow-up data. A sensitivity analysis prompted the limitation of the follow-up period to 2 years. Following this, a preliminary analysis was carried out to determine key potential predictors of mortality. In the final adjusted model, only covariates with a *p*-value of less than 0.20 were retained.

## Results

### Sample characteristics

During the 24-year period, a total of 110 patients were diagnosed with EC at AUBMC. The male to female ratio was 2.3. The mean age at diagnosis was 62.3 ± 12.1 years. The majority of patients were Lebanese (69%). The rest were Arabs from neighboring countries including Syria, Palestine, and Iraq. Out of these patients, 49% had esophageal squamous cell carcinoma (ESCC) and 51% had esophageal adenocarcinoma (EAC). The most common presenting symptoms were dysphagia (84%) and weight loss (64%) (Table 1). ESCC tended to occur more in the upper and middle esophagus (70.9%), while EAC occurred more at the level of the lower esophagus and gastroesophageal junction (88.7%). In addition, patients with EAC were more likely to be overweight or obese, whereas patients with ESCC were more likely to have a normal or low BMI (Table 2).

**Table 1:** Clinical characteristics of esophageal cancer patients diagnosed at AUBMC between 1995 and 2019

Factor		n (%)
Presenting signs and symptoms ^a^ (*n* = 101)	Dysphagia	85 (84.2%)
	Weight loss	62 (61.3%)
	Epigastric/Retrosternal pain	17 (16.8%)
	Anorexia	18 (17.8%)
	Odynophagia	12 (11.8%)
	GI bleeding	9 (8.9%)
	Persistent cough	8 (7.9%)
	Regurgitation	4 (4.0%)
	Hoarseness	3 (3.0%)
	Anemia	2(2.0%)
	Hepatomegaly	1(1.0%)
	Lymphadenopathy	1 (1.0%)
Histological type (*n* = 110)	ESCC	54 (49.1%)
	EAC	56 (50.9%)
Stage at diagnosis (*n* = 84)	I	8 (9.5%)
	II	12 (14.3%)
	III	50 (59.5%)
	IV	14 (16.7%)
Grade (*n* = 93)	Poorly differentiated	24 (25.8%)
	Moderately differentiated	61 (65.6%)
	Well differentiated	7 (7.5%)
	Undifferentiated	1 (1.1%)
Location of tumor (*n* = 101)	Upper esophagus	20 (19.8%)
	Middle esophagus	20 (19.8%)
	Lower esophagus	29 (28.7%)
	GE junction	32 (31.7%)

^a^ Total percentage greater than 100% as some patients presented with several symptoms

**Table 2:** Characteristics of esophageal cancer patients diagnosed at AUBMC between 1995 and 2019 by histological type

Factor	Variable	ESCC (*n* = 54)	EAC (*n* = 56)	*p* value
Age *n* = 110	< 50	16.7%	12.5%	0.786
	50–69	51.9%	57.1%	
	≥70	31.5%	30.4%	
Sex *n* = 110	Male	53.7%	87.5%	< 0.001
	Female	46.3%	12.5%	
BMI *n* = 84	Underweight	21.4%	2.4%	.005
	Normal range	45.2%	28.6%	
	Overweight	21.4%	50%	
	Obese	11.9%	14.3%	
Tobacco consumption *n* = 93	Current or past smoker	67.4%	44%	.024
Alcohol consumption *n* = 88	Yes	29.3%	21.3%	.388
Pre-existing esophageal disease *n* = 64	GERD	20.7%	28.6%	0.469
	Barrett’s	4.2%	21.4%	0.107
	Esophageal injury	6.9%	0%	0.153
Pre-existing gastric disease *n* = 64	Atrophic gastritis	30.8%	10.5%	0.055
	*H. pylori* infection	11.5%	10.5%	1.000
	Hiatal hernia	11.5%	10.5%	1.000
Tumor location *n* = 101	Upper esophagus	39.6%	1.9%	<.001
	Middle esophagus	31.3%	9.4%	
	Lower esophagus	25%	32.1%	
	GE junction	4.2%	56.6%	
Tumor location combined *n* = 101	Upper + Middle	70.8%	11.3%	<.001
	Lower + GE junction	29.2%	88.7%	
Tumor stage *n* = 54	I	7.7%	11.1%	.490
	II	20.5%	8.9%	
	III	54.6%	62.2%	
	IV	15.4%	17.8%	
Tumor grade *n* = 93	Poorly differentiated	19.1%	32.6%	.385
	Moderately differentiated	70.2%	60.9%	
	Well differentiated	8.5%	6.5%	

Most of our patients had advanced disease on diagnosis, where 76.4% had stage 3 or 4 and the majority (65.6%) had a moderately differentiated tumor.

### Risk factors

As shown in Table 2, patients in both types of EC were predominantly males. Notably, only one eighth of patients with EAC were women. Most patients with ESCC were current or past smokers at the time of diagnosis, whereas those with EAC were mostly nonsmokers. A minority of patients in both groups consumed alcohol. Most patients with ESCC had a normal BMI at diagnosis. By contrast, the majority of those diagnosed with EAC were overweight or obese.

Most of our EC patients had no documented significant pre-existing esophageal or gastric disease. Only a minority of patients had Barrett’s esophagus (BE), caustic injury or atrophic gastritis. BE (confirmed endoscopically or pathologically) was present in 21.4 and 4.2% of patients with EAC and ESCC, respectively.

### Survival data

Patients were followed up until death or last day of admission/clinic visit. The median survival rates among our esophageal cancer patients was 9 months (Table 3). Year 2010 was the year in which the introduction of the neoadjuvant chemotherapy/radiotherapy for the treatment of EC took place. When comparing our survival rates before and after 2010, we found no significant difference. Furthermore, no difference in survival was noted with respect to location or histologic type of EC. On the other hand, more advanced stage was associated with worse survival (*p* = 0.037). Only 13.6% of our patient were lost to follow up.

**Table 3:** Median survival of patients with esophageal cancer diagnosed at AUBMC between 1995 and 2019

Factor	Variable	Median survival in months (IQR)	*p* value
Date of diagnosis *n* = 97	< 2010	4 (1–17.8)	0.186
	> 2010	10 (4–32.5)	
Location *n* = 93	Upper + Middle	6.5 (2–13.5)	0.395
	Lower + GE junction	9 (3–30)	
Type *n* = 97	SCC	7 (2–25)	.803
	AC	9 (3–26.3)	
Tumor grade *n* = 85	Poorly differentiated	4 (1.5–22.5)	.851
	Moderately differentiated	9 (3–25.5)	
	Well differentiated	5 (1.8–41.3)	
Stage *n* = 76	Stage 1	45 (1–69)	.037
	Stage 2	3 (1.3–6)	
	Stage 3	12 (4–28)	
	Stage 4	6.5 (3–26.5)	

Median survival – months (IQR): 9 (3.0–25.5), *n* = 97 patients

### The neutrophil to lymphocyte ratio

We investigated the complete blood counts of our EC patients upon admission. As shown in Table 4, the mean NLR in our patients was 5.20 ± 6.8. We found no significant difference between EAC and ESCC, or between the different tumor grades or stages. The NLR had an area under the curve (AUC) for end of follow-up mortality of 0.560 (95% CI: 0.374–0.746, *p* = 0.488). On survival analysis, the first cox regression model which included age, gender, TNM staging and grading showed that NLR was a significant predictor of mortality, with a hazard ratio of 1.095 (*p* = 0.011). For the final cox regression analysis, a more rigorous statistical approach was done, and follow-up was capped at 2 years based on a sensitivity analysis. Crude analyses were first done to screen for variables that are associated with mortality, and only those that had a *p*-value of < 0.2 were included in the final model. In the adjusted model, an NLR greater than 3.5 was found to be a significant predictor of mortality. This means that patients with an NLR above this threshold had a statistically significant higher risk of mortality within the two-year period, with an adjusted hazard ratio of 5.059 (1.054–24.270), *p* = 0.043 (Table 5).

**Table 4:** Mean Neutrophil Lymphocyte ratio of esophageal cancer patients diagnosed at. AUBMC between 1995 and 2019

Factor	Variable	NLR^a^	*P*-value
Tumor type	Squamous cell carcinoma (*n* = 33)	5.9 (9.2)	0.420
	Adenocarcinoma (*n* = 37)	4.5 (3.4)	
Tumor stage	I (*n* = 4)	9.0 (14.2)	0.637
	II (*n* = 8)	3.7 (2.5)	
	III (*n* = 37)	4.8 (7.5)	
	IV (*n* = 10)	4.0 (3.1)	
Tumor grade	Poorly differentiated (*n* = 15)	4.8 (4.4)	0.530
	Moderately differentiated (*n* = 40)	6.2 (8.5)	
	Well differentiated (*n* = 6)	2.1 (0.4)	

^a^Values are produced as means (±SD)

Mean neutrophil lymphocyte ratio: 5.2 (6.8), *n* = 70 patients

**Table 5:** Predictors of mortality at 24 months in patients with esophageal cancer

	Unadjusted HR (95%Cl)	*P*-value	Adjusted^a^ HR (95%Cl)	*P*-value
Age at time of diagnosis	1.033 [0.996,1.071]	0.083	1.027 [0.972,1.085]	0.338
Tumor stage at diagnosis > 2	1.683 [0.383, 7.390]	0.490		
Anaplastic or high grade^b^	0.591 [0.173,2.018]	0.402		
Adenocarcinoma^c^	0.969 [0.427, 2.916]	0.939		
NLR > 3.5	4.511 [0.958,21.249]	0.057	5.059 [1.054,24.270]	0.043*

^a^ Cox regression model adjusted for age at time of diagnosis and neutrophil/lymphocyte ratio (NLR)

*HR* hazard ratio, *CI* confidence interval

^b^ Compared to intermediate (moderately differentiated) and low grade (well-differentiated)

^c^ Compared to squamous cell carcinoma

*Designates a significant difference between the two groups with *P*-value < 0.05

### Comparison of the incidence of esophageal cancer between the Middle East, east and west

As shown in Table 6, the incidence of EC in Middle Eastern (ME) countries ranged between 0.7 and 2.1 per 100,000 per annum in males and between 0.36 and 1.1 per 100,000 per annum in females. Lebanon had the lowest incidence rates, among all countries, in both sexes. The US, a representative of developed Western countries, had a relatively higher incidence rate than ME countries (4.8 and 1.1 per 100,000 per annum in males and females respectively). Asian belt countries, including China and Iran, had EC rank among the 10 most common malignancies whereby the incidence rates were 4.6–19.7 and 3.5–8.2 per 100,000 per annum in males and females respectively.

**Table 6:** Age- standardized incidence rates of esophageal cancer^a^ (per 100,000 per annum)

	Esophageal cancer incidence rates (per 100,000 per annum)		
	Males	Females	Average
US	4.8	1.1	3
China	19.7	8.2	13.9
Iran	4.6	3.5	8.1
Turkey	1.8	1	1.4
Lebanon	0.77	0.36	0.6
Syria	1.1	0.72	0.91
Palestine	1.4	0.64	1
Jordan	1.3	0.75	1
Kuwait	1.5	0.93	1.2
Iraq	1.2	1.1	1.1
Egypt	2.1	1.7	1.9
Cyprus	1.6	0.31	0.9

^a^ Data derived from Cancer Incidence in Five Continents (CI5 Vol X and CI5plus), 2020, maintained by the IARC (Lyon, France) and Globocan 2020 [1]

According to the National Cancer Registry database of the Ministry of Public Heath in Lebanon, the age standardized incidence of gastrointestinal malignancies had varied throughout the years (Table 7). Some gastrointestinal cancers such as pancreatic cancer registered an increased incidence from 3.4 to 5.6 per 100,000 per annum, while others kept stable rates such as gastric cancer. EC, in all cases, remained the tumor with the lowest incidence across the years reaching a maximum of 1.1 per 100,000 per annum in males in 2016.

**Table 7:** Age- standardized incidence rates of gastrointestinal malignancies in Lebanon (Per 100,000 per annum)^a^

		Esophagus	Gastric	Colon	Rectal	Liver	Gallbladder	Pancreatic
2003	Male	–	6.2	14.1	4.1	1.8	1.8	3.4
	Female	–	5.1	13.1	3.4	1.5	1.5	2.9
2008	Male	0.9	8.1	15.3	4.2	4	1.2	4.7
	Female	0.5	6.7	14.1	4.8	3.9	1.7	4.2
2012	Male	0.9	6.5	17.9	7.3	3.3	1.3	3.5
	Female	0.6	4.7	15	6.0	1.9	1.8	2.7
2016	Male	1.1	6.4	16.5	6.5	4.2	1.8	5.6
	Female	0.6	5.2	15.2	4.6	2.3	1.6	4

^a^ Data derived from the National Cancer Registry database of the Ministry of Public Heath in Lebanon

## Discussion

This is the first study that evaluates the epidemiology and the clinical profile of EC in Lebanon since the 1990’s. It is also the first to address the association between the NLR and survival in EC in Lebanon. It shows that EC, regardless of the histologic type, is very uncommon among the Lebanese population compared to the rest of the world and its incidence rates were reported to be among the lowest worldwide [5]. Furthermore, our data suggest that a higher NLR may be associated with worse survival in EC.

According to GLOBOCAN 2020 and the national database in 2016, the incidence of EC seems to be stable or even decreasing in Lebanon, in contrast to the global trend. Between 2003 and 2008, the incidence of esophageal carcinoma and melanoma decreased, that of gastric and pancreatic cancers increased, and that of colon cancer was stable [6].

New worldwide data suggest a shift in EC epidemiology. The incidence of EAC increased after 1978 in the West, and in 1994, it surpassed ESCC in the US [2]. Yet, ESCC is still the predominant histological subtype in the East, especially in the Asian belt [7]. Our study suggests that both ESCC and EAC are equally prevalent in Lebanon. A retrospective study performed in 1974 reported a ratio of ESCC to EAC of 1.3:1 in the ME compared to 11:1 in the Arabian Peninsula (Saudi Arabia, Kuwait, Qatar and Abu Dhabi) [8], possibly due to a higher intake of hot beverage like tea in addition to possible nutritional deficiencies [5]. The small number of patients in our study does not permit trending prevalence over time. However, it does not seem to mirror the shift in epidemiology that occurred in the West, or the predominance of ESCC in the East.

Smoking, obesity and GERD are recognized risk factors for ESCC and EAC [9]. For ESCC, other risk factors may include red meat consumption, alcohol, nass use (a chewing tobacco product), opium consumption, hot tea drinking, poor oral health, low intake of fresh fruits and vegetables, and low socioeconomic status [10]. In the West, factors that may have contributed to the decreasing incidence of ESCC are increased awareness, public education and anti-smoking and alcohol misuse campaigns [3]. We do not consider these to be contributing factors to Lebanon’s low incidence rates given the near absence of such campaigns, as well as the high rates of smokers with an average of 2000–3000 cigarettes smoked per person/year [11].

Equally interesting is the observation of a low prevalence of both BE and EAC in Lebanon despite a high prevalence of GERD and obesity. The principal hypothesis linking GERD and obesity to EAC is that chronic GERD, which is exacerbated by abdominal obesity, initiates the metaplasia-dysplasia-carcinoma sequence, and increase the risk of EAC through BE [12]. The prevalence of GERD was reported to be 24.8% among a representative sample of Lebanese in 2012 [13]. Likewise, obesity rate is increasing and reached 23.1% in 2014 as reported by the WHO. This is higher than that of China (7.4%), lower than that of the US (33.6%) and comparable to neighboring countries like Jordan (23.7%) and Turkey (22.2%) [14]. Biopsy proven BE, however, has a very low prevalence of 0.24% in unselected Lebanese undergoing endoscopy, compared to that of 1–2% in the West [15]. In our study, BE (endoscopically or histologically) was present in 10.7% of patients with EAC, much lower than the published rates in the literature. The low BE prevalence in Lebanon might be due the retrospective nature of our study, but it is consistent with recent reports by others [15].

Collectively, Lebanese have high smoking rate, high prevalence of GERD and obesity, but low prevalence of BE and low incidence of both ESCC and EAC. Genetic susceptibility may play a major role in EC. EC develops in very few exposed individuals [3]. Migrants from the ME to Australia were shown to have a lower cancer incidence than other Australians for many cancer types, including the esophagus [16]. Genetic studies uncovered associations between few genes and ESCC, such as the ECRG1 gene in Kashmiri population in India. Other genes include alcohol dehydrogenase 1B variants, phosphodiesterase 4D and the aldehyde dehydrogenase 2 which were reported in the Chinese and Japanese populations. These genes were associated with an increased risk of ESCC [3]. However, there are still limited genetic studies on EC in the West. ME countries, including Lebanon, may have a common “protective” genetic profile against EC despite the high rate of smoking, obesity and GERD. Other factors that may explain the low incidence of EAC include the Mediterranean/ME diet which is rich in vegetables and fruits, the high prevalence of *H. pylori* which is speculated to be protective, the wide use and availability of over-the-counter proton pump inhibitors (PPIs) and possible under-recognition of BE as suggested by the Asia-Pacific Barrett’s Consortium [15, 17].

The prognosis of EC is poor and is related to multiple factors including disease stage as shown in our study. Another factor may be the NLR. Neutrophils are an established part of peritumoral inflammatory cell infiltrate, named tumor-associated neutrophils (TANs). The infiltrate seems to be a direct result of cancer cell’s activity; hence the presence of these neutrophils is related to tumor growth (30). Multiple previous studies have mentioned the importance of NLR as an indicator of survival during the pre-operative stages of some cancers. In a study by Zhou et al., patients with higher NLR (> 5) at baseline had worse progression-free survival (PFS) and overall survival [18]. In our study, a cut-off of 3.5 was used, and the NLR had an adjusted hazard ratio of 5.1 with 2-year mortality, meaning that patients with an NLR exceeding 3.5 were more than five times as likely to die within 2 years compared to those with lower NLR values, after adjusting for other variables in the model. This significant finding suggests that a NLR could serve as a robust prognostic marker for mortality in this patient population, potentially aiding clinicians in identifying individuals at greater risk and guiding more targeted interventions.

There are several limitations to our study. The retrospective nature of data collection might lead to selection bias. Further, our patients might not be representative of the Lebanese population. Although AUBMC is a major tertiary referral center in Lebanon, people in the periphery of the country might not have access to medical care and some might not have a financial coverage to be treated at this center. Moreover, one third of our patients were from neighboring countries and not Lebanese. However, we think EC epidemiology in those countries is similar to that of Lebanon and is different from that of Western countries as shown in Table 6. In addition, there was a high rate of missing data. Patients may follow up at other hospitals or leave the country, which might have affected the accuracy of our survival estimates. Moreover, the relatively small sample size may have had a substantial impact on our results. Conventional predictors of mortality such as tumor grade and stage did not emerge as significant in our cox regression analysis, a finding that deviates from established clinical expectations. This could be due to the limited power of our study to detect differences, which is often constrained by the number of events in relation to the number of predictive factors being assessed. Moreover, a small sample sizes can lead to overfitting of the model and wide confidence intervals, which undermines the reliability of the hazard ratios estimated. Despite the limitations, we were able to identify important clinical information that were not captured nationally, including the prognostic importance of the NLR. Nonetheless, should we gain access to a more extensive dataset, such as one derived from a national cancer database, we could engage in a more nuanced analysis. With a larger cohort, the statistical power would be enhanced, thereby facilitating a more robust examination of the variables and a more reliable identification of true predictors of mortality among patients with esophageal cancer. Such enriched data could enable us to deliver more precise prognostic models, ultimately improving patient stratification and informing clinical decision-making.

In conclusion, both EAC and ESCC seem to be quite uncommon in Lebanon despite the high level of tobacco consumption and the high prevalence of GERD and obesity. In addition, elevated NLR may be associated with worse prognosis in EC patients.

## References

[1] Sung H, Ferlay J, Siegel RL, Laversanne M, Soerjomataram I, Jemal A, Bray F. Global cancer statistics 2020: GLOBOCAN estimates of incidence and mortality worldwide for 36 cancers in 185 countries. CA Cancer J Clin. 2021. 10.3322/caac.21660 33538338

[2] Feldman M, Friedman L, Brandt L (2016). Sleisenger and Fordtran's gastrointestinal and liver disease.

[3] Gupta B, Kumar N (2017). Worldwide incidence, mortality and time trends for cancer of the oesophagus. Eur J Cancer Prev..

[4] Jemal A, Bray F, Center MM, Ferlay J, Ward E, Forman D (2011). Global cancer statistics. CA Cancer J Clin..

[5] Freedman LS, Edwards BK, Ries LAG, Young JL (2006). Cancer incidence in four member countries (Cyprus, Egypt, Israel, and Jordan) of the Middle East Cancer consortium (MECC) compared with US SEER.

[6] Shamseddine A, Saleh A, Charafeddine M, Seoud M, Mukherji D, Temraz S (2014). Cancer trends in Lebanon: a review of incidence rates for the period of 2003-2008 and projections until 2018. Popul Health Metrics..

[7] Pennathur A, Gibson MK, Jobe BA, Luketich JD (2013). Oesophageal carcinoma. Lancet (London, England)..

[8] Razek MS, Nassar VH (1974). Carcinoma of the esopagus in the Middle East. Lebanese Med J..

[9] Charafeddine MA, Olson SH, Mukherji D, Temraz SN, Abou-Alfa GK, Shamseddine AI (2017). Proportion of cancer in a middle eastern country attributable to established risk factors. BMC Cancer..

[10] Zhang Y (2013). Epidemiology of esophageal cancer. World J Gastroenterol..

[11] Eriksen M, Mackay J, Schluger N, Islami F, Drope J (2015). The Tobacco Atlas.

[12] Long E, Beales IL (2014). The role of obesity in oesophageal cancer development. Ther Adv Gastroenterol..

[13] Awada S, Rachidi S, Al-Hajje A, Zeidan RK, Bou Kansour N, Abboud C (2014). Gastro-esophageal reflux disease in Lebanese adults: effects on quality of life and correlates. Pharmacologia..

[14] World Health Organization. Prevalence of obesity among adults, ages 18+, 1975-2016 (age standardized estimate): both sexes, 2016. In; 2016.

[15] Masri O, Ibrahim F, Badreddine R, Chalhoub JM, Sharara AI (2015). Prevalence of Barrett's esophagus in Lebanon. Turk J Gastroenterol..

[16] McCredie M, Coates M, Grulich A (1994). Cancer incidence in migrants to New South Wales (Australia) from the Middle East, 1972-91. Cancer Causes Control: CCC..

[17] Ho KY, Asia-Pacific Barrett's Consortium (2013). Is Barrett's esophagus an over-hyped disease in the west, and an underdiagnosed disease in the east?. Dig Endosc..

[18] Zhou XL, Li YQ (2017). Neutrophil-to-lymphocyte ratio as a prognostic biomarker for patients with locally advanced esophageal squamous cell carcinoma treated with definitive chemoradiotherapy. Sci Rep..

